# Parental Low Level of Education and Single-Parent Families as Predictors of Poor Control of Type 1 Diabetes in Children Followed in French Guiana

**DOI:** 10.3390/ijerph22071051

**Published:** 2025-06-30

**Authors:** Christelle Boyom Samou-Fantcho, Falucar Njuieyon, Nadjia Aigoun, Narcisse Elenga

**Affiliations:** 1 Centre Hospitalier de L’ouest Guyanais Franck Joly, Saint-Lauren-du-Maroni 97393, French Guiana; c.samou-fantcho@ch-ouestguyane.fr (C.B.S.-F.); n.aigoun@yahoo.fr (N.A.); 2 Centre Hospitalier de Cayenne, Cayenne 97306, French Guiana; falucar.njuieyon@chi-fsr.fr

**Keywords:** type 1 diabetes, predictors of poor control, children, parental low level of education, single-parent families, French Guiana

## Abstract

This study aimed to determine the prevalence of type 1 diabetes mellitus (T1DM) in French Guiana and describe the social profiles of the patients. We conducted a multicenter cross-sectional study of children under 18 years who were diagnosed with type 1 diabetes and followed up from 2002 to 2021. Over a 20-year period, 48 children under 18 years with type 1 diabetes living in French Guiana were included in the study, out of a total of 59 cases. There were 26 girls and 22 boys. The median age at diagnosis was 8.52 years [IQR 6–12]. The incidence rate was 5.9 per 100,000 people in children aged 0–18 years. The 5–9-year age group was the most affected 43.7% (95% CI 38–51%). Of these children, 56.2% (95% confidence interval 40–70%) lived in single-parent households, and 35% (95% CI 23–57%) of the parents had a primary education. Of the children, 29% (95% CI 21–42%) were from families with no resources. Diabetes was diagnosed by ketoacidosis in 56% (95% CI 38–74%) of the patients. Forty percent (95% CI 35–66%) of the patients had an HbA1c > 9%. There was an imbalance in the prevalence of children with higher Hba1c (>9%), with 18.7% (95% CI 10–29%, *p* < 0.001) of children whose parents had a low level of education having an Hba1c > 9% compared with only 6% (95% CI 3–10%) of children whose parents had a university degree, and a marked imbalance in the prevalence of children with High Hba1c (>9%) among children from single-parent families (22.9%, 95% CI 17–30%) compared with children whose parents lived in couples (8%, 95% CI 5–12%). The 10–14-year age group (18.7%, 95% CI 11–25%) had the highest imbalance in the prevalence of poor diabetes control between children whose parents had lower versus higher education levels. Diabetic retinopathy and diabetic nephropathy were the only reported complications. The multivariate analysis showed that a low level of parental education (Odds ratio 2.9 [95% CI 2.1–4.5], *p* < 0.001) and single-parent families (Odds ratio 3.1 [95% CI 2.6–4.3], *p* < 0.001) were predictors of poor control of T1DM. However, the lack of social insurance coverage at diagnosis was not associated with poor T1DM control (*p* = 0.4). In conclusion, these sociodemographic factors should be considered when caring for children with T1DM in French Guiana.

## 1. Introduction

Type 1 diabetes mellitus (T1DM) is the most common chronic endocrine disease in childhood, and its incidence is increasing worldwide, likely due to epigenetic and environmental factors [1]. It is a serious chronic disease resulting from the autoimmune destruction of insulin-producing cells in the pancreas. Although previous research has elucidated the HLA genotypes that predispose to T1D, it is thought that a combination of genetic predisposition and environmental factors (viruses, microbiological factors, stress, etc.) likely contributes to its development [1]. Worldwide, it accounts for 5–10% of all diabetes cases, 90% of which are in children. In France, the prevalence is estimated at 19.1 per 100,000 inhabitants [2]. Moreover, 2500 cases of type 1 diabetes were recorded in children aged between 6 months and 14 years. In French Guiana, the prevalence was 3.6 per 100,000 inhabitants between 2013 and 2015 [3]. The prevalence of T1DM has also increased in low- and middle-income countries [4]. T1DM has a negative impact on the physical and mental health, emotional development, and vital prognosis of affected children and adolescents. According to Erikson’s developmental theory, the disease causes significant changes in the development of children and adolescents with T1DM [5]. Clinically, T1DM is characterized by the cardinal syndrome of polyuria, polyphagia, polydipsia, and weight loss. There is also a break in the weight curve, secondary enuresis, and diabetes ketoacidosis (DKA) in 48% of cases. It is characterized by chronic hyperglycemia with fasting blood glucose > 126 mg/dL, blood glucose > 200 mg/dL at any time and the presence of autoantibodies (anti-islet cell, anti-GAD, anti-IA2, anti-insulin, and anti-ZnT8) [6]. If not properly managed, this disease can lead to serious complications.

Many studies confirm the importance of socio-demographic factors in the care of children with T1DM [7,8,9]. In French Guiana, social security covers the cost of care, which is organized equally and free of charge for all. Despite a well-organized healthcare system and the widespread availability of advanced diabetes management technologies, only one-third of adult patients in our region achieve their glycemic targets [10]. It is therefore imperative to study the socio-demographic factors likely to influence glycemic control in the pediatric population of a modern healthcare system so that we can better target support to the families who need it most and thus help more children achieve optimal metabolic control of their diabetes.

The aim of this study was to determine the prevalence of pediatric T1DM in French Guiana and to describe the social profile of the patients.

## 2. Methods

### 2.1. Study Setting

French Guiana is a French territory located in South America between Suriname to the west and Brazil to the east. Its population (286,618 inhabitants in 2021) is ethnically diverse and includes Amerindians, blacks, whites, Hispanics, Asians, and mestizos [11]. Approximately 32.6% of the population is aged between 0 and 14 years. There is a high level of vulnerability, with a poverty rate of 34–74% [12]. The unemployment rate was 32% (for the 15–64 age group). Only 32.8% of the population over the age of 18 years attended school. Of the working population, 47.4% had no qualifications and 13.2% had a General Certificate of Secondary Education. Furthermore, 44% of families were single-parent families (38% of women lived alone with their children). There is massive immigration from neighboring countries [13].

### 2.2. Study Design

This was a cross-sectional study. The study population consisted of children under the age of 18 who were diagnosed and followed up by a pediatric diabetes specialist in French Guiana between 1 January 2002 and 31 December 2021. In addition to information from medical records, children and their parents were invited to participate in the study and complete a questionnaire designed to collect basic information about the family. This questionnaire was administered during a regular follow-up visit to a consultation room or a children’s day clinic in hospitals in French Guiana. The interval between visits was generally 3 to 6 months, depending on the case.

#### 2.2.1. Data Collected

The data collected were:

Clinical data: age, sex, age at diagnosis, treatment regimen, follow-up, HbA1c, complications (retinopathy, nephropathy), other associated autoimmune diseases, etc.

A questionnaire (see Supplementary Material) was used to collect certain socio-demographic data: employment, parental education, family situation, and social security coverage. The questionnaire was completed by each patient three to six months after their diagnosis.

Definitions: In line with recommendations from the French authorities, we have defined ‘regular follow-up’ as involving two consultations with a pediatric diabetes specialist and two HbA1c tests each year. The quality of the follow-up depended on whether two or more visits were carried out within a year. Follow-up was irregular in cases involving fewer than two visits per year [10].

#### 2.2.2. Data Analysis

Data were entered into an Excel 2016 database and then anonymized. STATA 16.0 software (Stata Corp LP, College Station, TX, USA) was used for all descriptive analyses. The first and third quartiles and the median were used to classify quantitative variables according to statistical criteria. Student’s *t*-test was used for quantitative variables and the CHI2 test or Fisher’s exact test for qualitative variables. Logistic regression was performed to examine covariates and their associations with control of diabetes measured by HbA1c, based on the crude odds ratio (OR) and its confidence interval (CI). If the *p*-value was less than 0.05, the result was considered to be significant.

### 2.3. Ethics and Consent

This study was based on existing healthcare data and a questionnaire. All parents and patients were personally informed and asked to provide consent to participate in the study. All participants who were minors provided written informed consent from their parents or legal guardians. All data were extracted from the hospital databases after certification of the patients’ or parents’ written non-opposition. The study was approved by the ethics committee of the Centre Hospitalier de Cayenne under the number 0013/2022. However, parents and patients were informed of the use of their data by an information poster in all pediatric units. We also obtained parents’ consent to publish their children’s clinical data. Data were anonymized according to the French legislation (Loi no. 78-17 du 6 janvier 1978 relative à l’informatique, aux fichiers et aux libertés modifiée). According to the European regulation, French observational studies from data obtained routinely from patient healthcare records do not need the approval of an ethics committee [14,15].

## 3. Results

Over a period of 20 years, we recorded 59 children under the age of 20 years with T1DM in French Guiana. Eleven patients were excluded because their main residence was not in French Guiana (they were children of tourists or vacationers). Data from 48 patients living in French Guiana were included in the study. There were 26 girls and 22 boys.

### 3.1. Epidemiological Data

Based on all the data, the prevalence in 2021 was 5.9 per 100,000 population in children aged 0–18 years. The annual number varies from one case per year in 2002 to 10 cases per year in 2021. Therefore, it has increased significantly over the years. The median age at diagnosis was 8.52 years [IQR 6–12]. The 5–9-year age group was the most affected (43.7% (95% CI 38–51%).

### 3.2. Social Data

In French Guiana, 56.2% (95% CI 40–70%) of the children with diabetes lived in single-parent families. Poor glycemic control was greater in single-parent families (36.6%, 95% CI 25–55%, *p* < 0.001) than in children whose parents lived with a couple (13%, 95% CI 9–21%). Moreover, 35% (95% CI 23–57%) of parents of children with T1DM have primary education. Of the children, 29% (95% CI 21–42%) came from families with no resources or living with assistance (relatives or associations), 20.8% (95% CI 15–29%) had no social security coverage at the time of diagnosis, and 66.6% (95% CI 50–80%) had regular follow-up compared to 29.6% 95% (CI 21–40%) who had irregular follow-up. The follow-up was more irregular if the parents had a low level of education (*p* < 0.001). However, having a job did not influence the quality of follow-up (*p* = 0.1). Table 1 summarizes the socio-demographic and clinical characteristics of patients according to HbA1c levels.

### 3.3. Clinical Data

**Figure 1:**
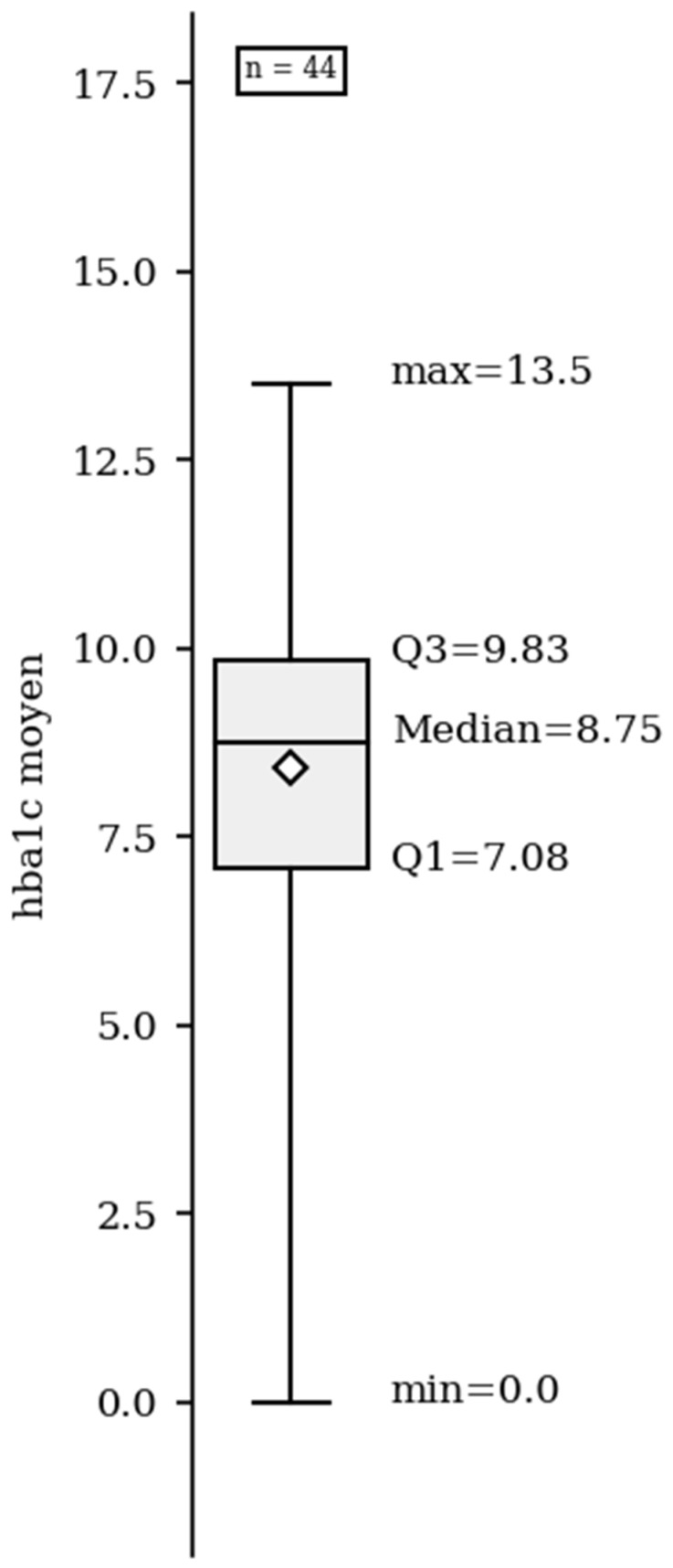
HbA1c median values.

Diabetes was detected as DKA in 56% (95% CI 38–74%) of patients, which was more common than polydipsia in 25% 95% (CI 19–32%) of patients (Table 2). We advocate that a satisfactory target for glycemic hemoglobin (HbA1c) level should be below 7.5%, but glycemic control in our cohort was unsatisfactory, with a median HbA1c level of 8.7% [7.8–9.83%] (Figure 1). Only 25% (95% CI 18–33%) of the patients had HbA1c levels within the target range. Moreover, 63% (95% CI 45–80%) of our patients had HbA1c levels outside the target range, while 40% (95% CI 35–66%) of the patients had an HbA1c > 9%.

**Figure 2:**
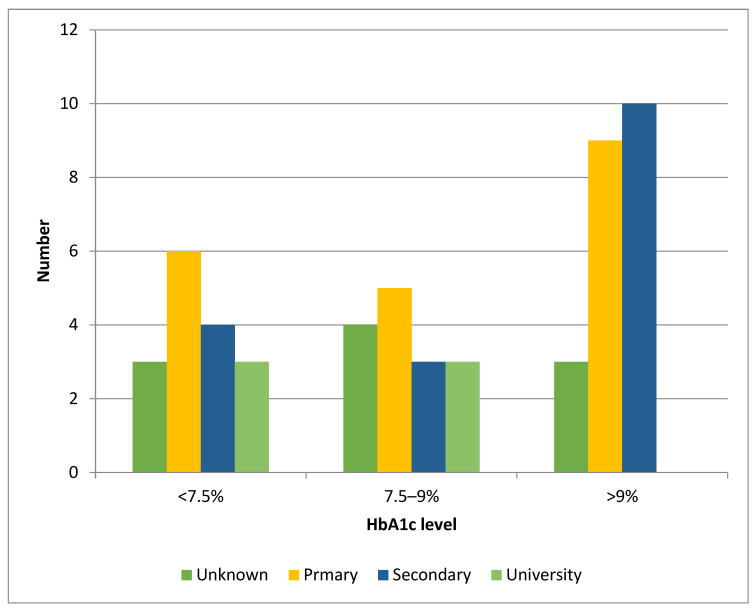
HbA1c level and parental level of education.

**Figure 3:**
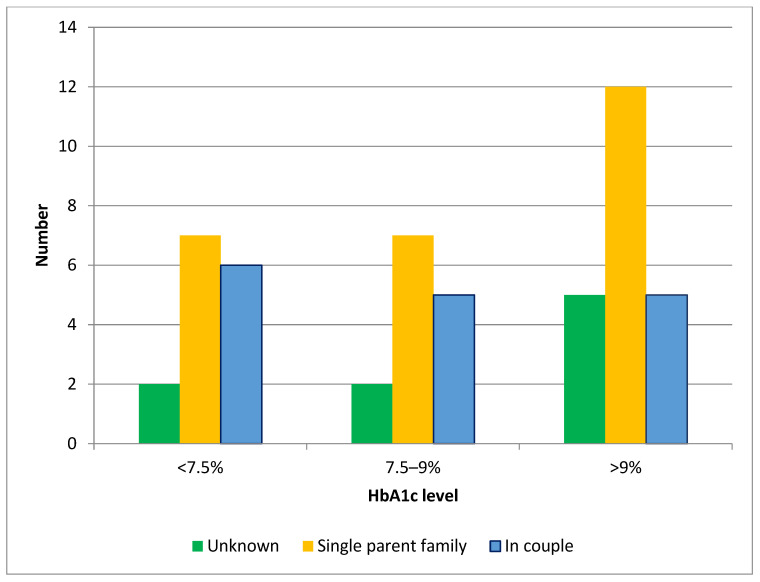
HbA1c levels and single-parent families.

There was an imbalance in the prevalence of children with higher HbA1c (>9%), with 18.7% (95% CI 10–29%, *p* < 0.001) of children whose parents had a low level of education having an Hba1c >9% compared with only 6% (95% CI 3–10%) of children whose parents had a university degree (Figure 2), and a marked imbalance in prevalence of children with high HbA1c (>9%) among children from single-parent families (22.9%, 95% CI 17–30%) compared with children whose parents lived in couples (8%, 95% CI 5–12%) (Figure 3). The 10–14-year age group (18.7%, 95% CI 11–25%) had the highest imbalance in the prevalence of poor diabetes control between children whose parents had lower versus higher education levels. Furthermore, 92% (95% CI 80–99%) of our patients were treated with a multi-injection basal-bolus insulin regimen, compared with 8% (95% CI 6–11%) using an insulin pump.

Of the patients, 45.8% had home nurses. The nurses came three times every day to perform a glucose finger-prick test and administer insulin. This was because it was difficult for the family to check the blood glucose levels and administer treatment. No other autoimmune diseases were found in any of the patients at the time of diagnosis. Diabetic retinopathy and diabetic nephropathy were the only reported complications. Among our patients, 4% (95% CI 2–6.5%) had had diabetes for at least 10 years. No complications were observed in any of these patients.

The multivariate analysis showed that parental low level of education (Odds ratio 2.9 [95% CI 2.1–4.5], *p* < 0.001) and single-parent families were predictors of poor control of T1DM (Odds ratio 3.1 [95% CI 2.6–4.3], *p* < 0.001). However, a lack of social insurance coverage at diagnosis was not associated with poor T1DM control (*p* = 0.4).

## 4. Discussion

The incidence of T1DM in our study population was 5.9 cases/100,000 inhabitants by 2021. The incidence of T1DM in the pediatric population has been rising steadily for several years, with potentially significant public health implications in the near future. These patients require early and effective glycemic control to prevent long-term complications [16,17,18,19]. Compared with mainland France [16], Brazil [20], and the French West Indies [16], our incidence rate appears to be underestimated. This could be explained by the retrospective nature of our study, which could lead to recruitment bias. Women predominated in our study, and this result is not consistent with that of previously published studies [21].

The median age at diagnosis was 8.52 years, confirming data showing that most children are diagnosed before the age of 10 [22]. Several studies have found a higher rate in the under-4 years age group and the 10–14-year age group [23,24]. DKA, a life-threatening complication of T1DM, was observed in 56% of cases. The frequency of DKA at T1DM onset varies from 12.8% to 80% worldwide [25]. The lowest rates are found in Canada and Scandinavian countries, while the highest rates are found in countries with a lower development index [25]. There is evidence of an association between increased awareness of the disease and DKA. Unfavorable socio-economic conditions increase the risk of DKA at disease onset [26]. How can this complication be detected early in children with type 1 diabetes? The onset of diabetes is preceded by a variable period of symptoms, including weight loss, polyuria, and polydipsia. Early treatment with insulin can prevent DKA. Therefore, primary-care professionals need to be trained, and the public needs to be made aware of the warning signs of T1DM in children so that these children can be referred quickly before the onset of DKA [26]. In our study, the mean HbA1c level was 8.7%, reflecting poor glycemic control. A similar rate was found by Choleau et in 2011 [24]. However, the independent predictive factors of high HbA1c were low parental level of education and single-parent families. The literature has identified several factors associated with high HbA1c levels, including adolescence, low socioeconomic status, low parental education, single-parent family structure, diabetes-related family conflict, lower parental involvement in diabetes care, and lack of a regular diabetes care provider [7,27,28,29]. Most of our children (56.2%) lived in single-parent families. This reflects French Guianese society, where 44% of families are single-parent families [30]. The lower the socioeconomic level or education level, the higher the HbA1c level. This result is consistent with previous studies [7,8,9]. Twenty-three percent of our patients had no regular follow-up, including those whose parents had a low level of education. Similar trends have been reported in the literature. A low level of education is a risk factor for poor follow-up and glycemic control [31,32]. On the other hand, whether the parents were employed did not affect follow-up. This finding is similar to that of the Gomes study in Brazil [32]. In our study, the absence of social security coverage at diagnosis did not affect the T1DM control. This could be explained by the fact that care is free for the chronically ill and even for those without social insurance. Unfortunately, the notion of diabetes-related family conflict was not investigated in our study.

The dominant insulin therapy regimen in our study was a multi-injection system. This differs from the insulin pump, which is currently the most widely used model in other regions (49.9% in Aquitaine) [33]. Data in the literature show that the insulin pump has several advantages [34,35]: (i) reduced number of complications, (ii) best method of mimicking physiological insulin secretion, (iii) method of choice for children under the age of 6, (iv) improved quality of life by reducing the number of injections and severe hypoglycemia, and (v) improved night-time control (blood glucose levels at bedtime, in the middle of the night, and on rising) compared with a regimen involving multiple injections of insulin. This highly specialized technique requires rigorous education and numerous precautions [34,35]. Several factors may explain why the pump is almost not used in French Guiana: the low level of education of parents, lack of trained staff, and reluctance of families. In addition, there are few pediatric endocrinologists in Guyana. However, we believe that with the help of nurses trained in diabetes education, many more children with diabetes will be able to benefit from insulin pump prescriptions. However, this prescription needs to be closely monitored to avoid malfunctions that require frequent medical consultations. In summary, our study shows that, as has previously been observed among adults, children with diabetes in French Guiana are not receiving adequate care. The main factors contributing to this situation are poor living conditions, a low density of healthcare professionals, and geographical isolation. Although these parameters are comparable to those in low-income countries, French Guiana has the advantage of having free healthcare as part of France and Europe. Local initiatives involving health mediators or nurse practitioners who are familiar with the local culture and are trained to assist with monitoring and treatment compliance could improve diabetes management for children in French Guiana.

### Limitations of Our Study

We acknowledge certain limitations of our study. Firstly, our study is subject to an inherent risk of selection bias because it relies solely on retrospective data collected from hospital records and questionnaires completed during routine follow-up appointments. Secondly, certain intimate questions, particularly about economic resources, were not answered. As we did not conduct a clinical trial or prospectively enroll and follow patients, we had limited control over potential confounding factors that could affect the validity of the associations we identified. Therefore, our results should be interpreted with caution. Nevertheless, we believe that the results are at least suggestive and useful for clinical practice.

## 5. Conclusions

Our study confirms the importance of considering socioeconomic factors in the management of childhood diabetes. The lowest socio-demographic markers in the family, such as parental education and living in a single-parent family, were associated with poor control of T1DM in children. It remains to be seen whether this is due to lower levels of parental knowledge about diabetes or lower levels of child perception of and compliance with diabetes control. These sociodemographic factors should be considered when caring for children with T1DM in French Guiana.

## Figures and Tables

**Table 1:** Socio-demographic characteristics of patients according to HbA1c levels.

Variables	HbA1c ≤ 7.5	HbA1c > 7.5	OR [95% CI]	*p*
	*N* = 12	*N* = 36		
Age at diagnosis (Median, IQR)	8.4 [6–11]	8.72 [6–13]		0.4
Gender, Female, *n* (%)	7 (58)	19 (53)		0.5
Origin				
African Caribbean	9 (75)	27 (75)		0.5
Hispanic	2 (17)	6 (17)		0.5
Caucasian	1 (8)	3 (8)		0.5
Parental level of education				
Primary education	2 (17)	10 (28)	2.1 [1.8–4.3]	<0.001
Secondary	3 (25)	16 (44)		0.1
University	7 (58)	8 (22)	0.7 [0.5–0.9]	<0.001
Unknown	0	2 (6)		
Parental marital status				
Single parent family	5 (42)	25 (69)	3.5 [2.5–4.6]	<0.001
In couple	7 (58)	11 (31)	0.6 [0.6–0.85]	<0.001
Poor parental financial status	4 (33)	10 (28)		0.4
Social security coverage	12 (100)	17 (47)		0.4

A bivariate logistic regression analysis was performed on the socio-demographic characteristics of patients according to their HbA1c levels.

**Table 2:** Clinical characteristics of patients according to HbA1c levels.

Variables	HbA1c ≤ 7.5	HbA1c > 7.5	OR [95% CI]	*p*
	*N* = 12	*N* = 36		
Circumstances of diagnosis				
Polydipsia	5 (42)	6 (17)	0.5 [0.4–0.7]	<0.001
Ketoacidosis	7 (82)	21 (58)		0.1
Unknown	0	9 (25)		
Follow-up				
Regular	12 (100)	20 (56)	0.6 [0.4–0.86]	<0.001
Irregular	0	14 (39)		
Unknown	0	2 (5)		
Chronic complications				
Retinopathy	0	1 (3)		
Diabetic nephropathy	0	1 (3)		
Treatment				
Insulin regimen	11 (92)	33 (92)		0.1
Insulin pump	1 (8)	3 (8)		0.1

A bivariate logistic regression analysis was performed on the clinical characteristics of patients according to their HbA1c levels.

## Data Availability

The data that support the findings of this study are available upon reasonable request.

## Supplementary Materials

The following supporting information can be downloaded at: https://www.mdpi.com/article/10.3390/ijerph22071051/s1, File S1: Survey Form.

## Abbreviations

T1DM	Type 1 diabetes mellitus
DKA	Diabetes ketoacidosis
HbA1c	Glycemic hemoglobin
