# Supplementary material for: Parental Low Level of Education and Single-Parent Families as Predictors of Poor Control of Type 1 Diabetes in Children Followed in French Guiana
Source: Int J Environ Res Public Health. 2025 Jun 30;22(7):1051. doi: 10.3390/ijerph22071051 (PMC12294541; doi:10.3390/ijerph22071051)
Supplement: Supplementary file 1 [file ijerph-22-01051-s001.zip › ijerph-3665673-supplementary.pdf]

SURVEY FORM

Family name : First name :

Date of birth : Age : Sex 1) M 2)F

Place of birth : 1) French Guiana 2) Outside French Guiana

Address :

Family environment

	Father	Mother
Surname and first name		
Date of birth		
Profession		
Nationality<br>Origin		
Medical and surgical history		
Address		
Telephone number		

Parents' marital status: 1)married 2)cohabiting 3)single parent 4/ unknown

Parents' level of education: 1)primary school 2)secondary school 3)university 4/ unknown

Number of people in the household :

Number of children :

Understanding of French language: 1) yes 2) not at all

Social aspects

Social security cover at diagnosis : 1)general scheme - 2)CMU - 3)AME 4)no cover

Distance between home and care facility: 1)less than 20km 2)20-30km 3)more than 30km

Private transport: 1)yes 2)no

Resources: 1)salary 2)benefits - assistance 4)no resources

Regular administrative situation: 1)yes 2)no

Some clinical aspects

Diabetes diagnosis year:

Place of diagnosis: French Guiana 1/yes 2/no

Age at diagnosis: 1) 0-4y 2)5-9y 3)10-14y 4)15-19y

Mode of discovery: 1)ketoacidosis 2)polyuropolydipsic syndrome 3)chance discovery

Comorbidities: 1)yes 2)no If yes, please specify :

Year of diagnosis :

HbA1C at diagnosis: % (in %)

Average HbA1C per year: % (in %)

Insulin therapy regimen: 1) pump 2)multiple injections

Home nurse: 1)yes 2)no

Insuline doses: 1) fixed 2) adjusted

Re-hospitalisation in the year of diabetes diagnosis: 1)yes 2)no

Reason for Re-hospitalisation (if yes): 1)imbalance 2)ketoacidosis 3)hypoglycaemia

Complications: 1)yes 2)no If yes, please specify :

Regular follow-up (every 3 months) 1) yes 2) no
